# Expression of toll‐like receptors and their regulatory roles in murine cardiac telocytes

**DOI:** 10.1111/jcmm.14416

**Published:** 2019-06-24

**Authors:** Shinan Li, Xiaokun Shen, Shaoheng He

**Affiliations:** ^1^ Institute for Translation Medicine Jinzhou Medical University Jinzhou China; ^2^ Allergy and Clinical Immunology Research Centre the First Affiliated Hospital of Jinzhou Medical University Jinzhou China

**Keywords:** immunosuppression, secretory profile, telocytes, toll‐like receptors

## Abstract

Telocytes, newly discovered in the last decade, are interstitial cells found in numerous organs, with multiple proposed potential biological functions. Toll‐like receptors (TLRs) play an important role in innate and adaptive immunity by recognizing pathogen‐associated molecular patterns (PAMPs). However, it is still unknown whether telocytes express these innate receptors. We sought to determine the expression and role of TLRs in telocytes. In our study, we primarily detected TLR1‐9 expression in telocytes. The proliferation, apoptosis and immunoregulatory activity of telocytes activated with or without TLR ligands were determined. Our results showed that purified telocytes expressed TLR2, TLR3 and TLR5. In particular, telocytes expressed high levels of TLR2 as observed using flow cytometry. When we stimulated telocytes with TLR2 or TLR3 agonists (Pam3CSK4, PolyI:C), iNOS expression was greatly increased after Pam3CSK4 treatment. Additionally, telocyte proliferation was reduced and cell apoptosis was increased after TLR agonist stimulation. A co‐culture experiment showed that supernatant from telocytes pretreated with Pam3CSK4 inhibited T cell activation much more than that from untreated telocytes and this effect was mediated by iNOS. Overall, our results demonstrated TLR expression on telocytes for the first time and provided evidence of an immunoregulatory role of telocytes, indicating their clinical potential.

## 1 INTRODUCTION

Telocytes were newly identified as unique interstitial cells that are distinguished from other interstitial cells of Cajal (ICC) and interstitial cells by their 2‐5 very thin body prolongations with a moniliform aspect.^1^ Telocytes were reported to exist in various cavitary and non‐cavitary organs, including heart, lung, liver, kidney, etc.^2^, ^3^, ^4^, ^5^ Many groups demonstrated that the 3D network, which is made by telocyte homo‐ or hetero‐cellular contacts, provides structural support between telocytes and other cells. Presumably, this support might contribute to tissue homeostasis, regeneration or repair and transmission of intercellular signals.^6^, ^7^, ^8^ Ultrastructural evidence showed that telocytes were spatially close not only to capillaries, nerve fibres, myocytes, smooth muscle cells and stem cells^9^, ^10^, ^11^ but also to macrophages, mast cells, eosinophils and lymphocytes.^12^, ^13^, ^14^, ^15^ Telocytes might modulate the immunocytes through direct synapse‐like contact^15^ or indirect paracrine communication by secreting soluble mediators, such as IL‐6, VEGF and nitric oxide.^11^, ^16^ Although telocytes were reported to be involved in multiple autoimmune, inflammatory and fibrotic disorders,^12^, ^17^ their number was decreased and their morphology was disrupted in these conditions.^18^ Thus, how telocytes respond and how their secretory profile is changed during stress remains unclear.

TLRs are important sensors in innate and adaptive immunity by recognizing exogenous pathogen‐associated molecular patterns (PAMPs) and endogenous molecules. A total of 12 TLR family members were found in mice and 10 were found in humans.^19^ TLRs are widely expressed not only on immune cells^20^, ^21^ but also on non‐immune cells, including haematopoietic stem and progenitor cells (HSPCs),^22^ endothelial cells,^23^ mesenchymal stem cells^24^ and fibroblasts.^25^ Although TLR activation has been recognized as boosting immune defense, researchers came to realize that TLR ligands could influence the proliferation, apoptosis and differentiation of non‐immune cells. TLR2 was expressed on intestinal and mammary epithelial cells. Loss of TLR2 in intestinal epithelial cells led to reduction of tumour formation. Moreover, TLR2 agonists could promote cell colony growth in vitro.^26^ Chen and colleagues reported for the first time that TLR3 and TLR4 were more highly expressed on human umbilical cord mesenchymal stem cells (UC‐MSCs), activation of which could affect the proliferation and adipogenic differentiation of UC‐MSCs.^27^ Telocytes were similar to MSCs to some extent.^28^ The discovery of TLRs on MSCs led us to explore the effect of TLRs on telocytes, which was still unclear until now.

In our study, we examined the expression of TLRs on cardiac telocytes and evaluated the function of TLRs on regulation of telocytes with TLR ligands. Our data showed that TLR2, which was abundant on telocytes, could significantly enhance the suppressive activity of telocytes on T cell activation.

## 2 MATERIALS AND METHODS

### 2.1 Isolation and culture of cardiac telocytes

Six‐week‐old male BALB/c mice (Beijing Vital River Laboratory Animal Technology Co., Ltd) were used to harvest hearts, which were placed in 1× PBS supplemented with 1% penicillin and streptomycin (PS). The hearts were minced into 1 mm^3^ pieces in a sterile culture dish containing Dulbecco's modified Eagle's medium (DMEM)/F12 (C11330500BT, Gibco; Thermo Fisher Scientific, Inc, China) supplemented with 1% PS. The pieces were washed by centrifugation at 300*g* for 5 minutes and were resuspended in PBS to remove the blood. An enzymatic digestion medium (collagenase II, Sigma‐Aldrich, 2 mg/mL) was added and the mixture was incubated at 37°C on a shaker for 1 hour. 1× PBS was required to terminate digestion. The solution was filtered through a 70 μm nylon mesh (EMD Millipore) and the collected suspension was centrifuged at 300*g* for 10 minutes. The cells were then seeded into 25 cm^2^ culture dishes containing DMEM/F12 supplemented with 10% foetal bovine serum (FBS; 10099‐141; Gibco; Thermo Fisher Scientific, Inc) and 1% PS and cultured at 37°C for 1.5 hours to allow fibroblast attachment. The unattached cells (almost telocytes) were replated onto a new dish with the above medium and the medium was replaced 24 hours later. Cells were selected, purified and further amplified for further experiments. Cell cultures were examined using an inverted microscope and photographed at different time‐points after seeding.

### 2.2 Flow cytometry

When purified telocytes had grown to 80% confluence, cells were detached by digestion in 0.25% trypsin/EDTA (Invitrogen, Thermo Fisher Scientific, Inc) within 1 minute. Then, cells were harvested for flow cytometry. The antibodies were used as follows: PE‐anti‐CD34, ‐TLR2, ‐TLR3, ‐PD‐1; APC‐anti‐CD3, ‐CD140a (PDGFR‐α); and Alexa Fluor^®^ 488 anti‐CD8α. All antibodies were purchased from BioLegend. Data were collected on a FACSVerse flow cytometer (BD Biosciences) and analysed using FlowJo software (TreeStar, Inc).

### 2.3 Immunofluorescent staining

Immunofluorescence staining was performed as described.^29^ The primary antibodies (armenian Hamster anti‐CD34, rat anti‐CD140a) were purchased from BioLegend. The secondary antibodies (DyLight™ 594 Goat anti‐hamster IgG, FITC‐Conjugated Goat anti‐Rat IgG) were purchased from Biolegend and ZSGB‐Bio respectively.

### 2.4 TLR activation of telocytes

When telocytes grew to 80% confluence, the supernatant was discarded and fresh medium (5% FBS, Gibco; DMEM/F12) was added into the system. Telocytes were stimulated with the TLR2 agonist Pam3CSK4 (3 μg/mL, Invivogen) or the TLR3 agonist PolyI:C (5 μg/mL, Invivogen) for 24 hours. Then, the supernatants were collected for further experiments.

### 2.5 Apoptosis assay

Telocytes treated as above were collected for the apoptosis assay. An Annexin V‐FITC/PI kit (BD Pharmingen) was used to stain cells for 15 minutes protected from light following the instruction. Flow cytometry was applied to detect cell apoptosis immediately. The TUNEL Apoptosis Assay Kit was purchased from Beyotime (Shanghai, China) and performed following the manufacturer's instructions.

### 2.6 RT‐PCR

Total RNA was isolated from telocytes and fibroblasts and real‐time qPCR was performed. Primer information is provided in the Supplementary Materials and Methods.

### 2.7 ELISA

ELISA kits for IL‐6, VEGF, TNF‐α, MCP‐1 (DAKEWE) and iNOS (Wuhan EIAab Science Co., Ltd.) were used to quantify cytokines.

### 2.8 T cell suppression assay

Supernatants of telocytes were collected after Pam3CSK4 stimulation with or without iNOS inhibitor for 24 hours. Lymphocytes (2 × 10^5^) from normal spleen activated by anti‐CD3/CD28 (10 mg/mL anti‐CD3; 5 mg/mL anti‐CD28) were co‐cultured with different supernatants as indicated above for 4 days in a round‐bottomed 96‐well plate at 37°C in 5% CO_2_. A functional CD8^+^ T cell suppression assay was performed by evaluating PD‐1 expression on CD8^+^ T cells.

### 2.9 Statistical analysis

All statistical analyses were performed with an unpaired Student's *t* test. The data are expressed as the mean ± SEM and differences were considered statistically significant when *P* < 0.05 (*P < 0.05; **P < 0.01; ***P < 0.001).

## 3 RESULTS

### 3.1 Identification of cardiac telocytes

**Figure 1:**
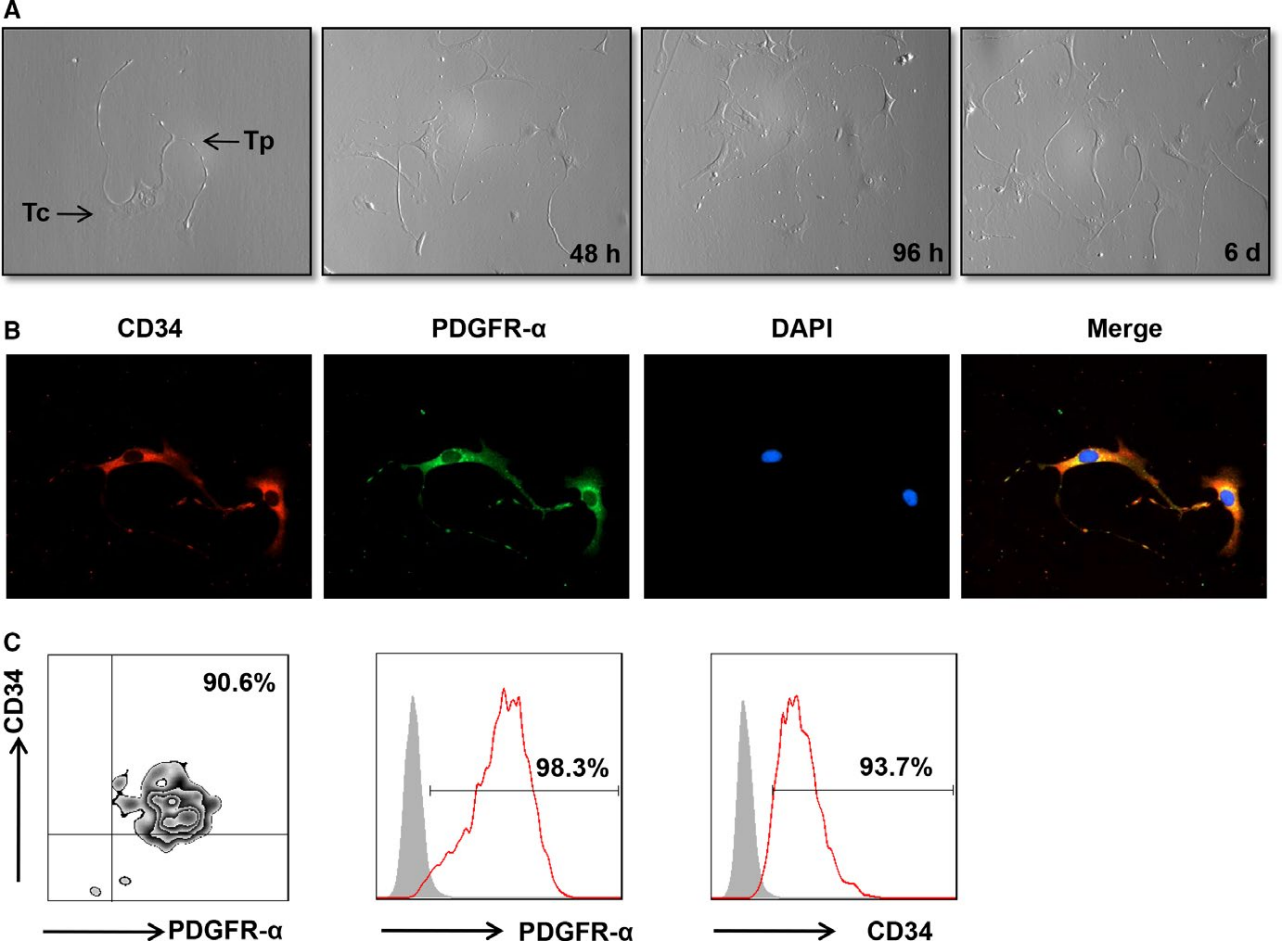
The identification of telocytes in the heart. (A) The representative morphology of telocytes under a light microscope. The images were collected at 48 hours, 96 hours and 6 days after fresh isolation. (B) Immunofluorescence staining for CD34 (red) and PDGFR‐α (green) with DAPI for nuclei. (C) The expression of PDGFR‐α and CD34 on purified telocytes were examined using flow cytometry

To examine the expression of TLRs on telocytes, we first attempted to isolate purified telocytes. As described in the methods, telocytes were purified after a repeated selection process. Telocytes were identified via morphology after primary cell culture at different time‐points (Figure 1A). Telocytes are characterized by a small cell body (Tc), the presence of extremely long and thin prolongations (telopode, Tps) and a significant moniliform aspect with many dilations along the telopode. Also, immunofluorescence staining was used to observe the morphology of telocytes. Cardiac telocytes were double positive for CD34/PDGFR‐α with moniliform telopodes (Figure 1B). Next, we used anti‐CD34/PDGFR‐α to identify the immunophenotype of telocytes using flow cytometry analysis. As shown in Figure 1C, cardiac telocytes expressed high levels of the two markers, indicating that the isolated telocytes were highly purified.

### 3.2 TLR expression of telocytes

**Figure 2:**
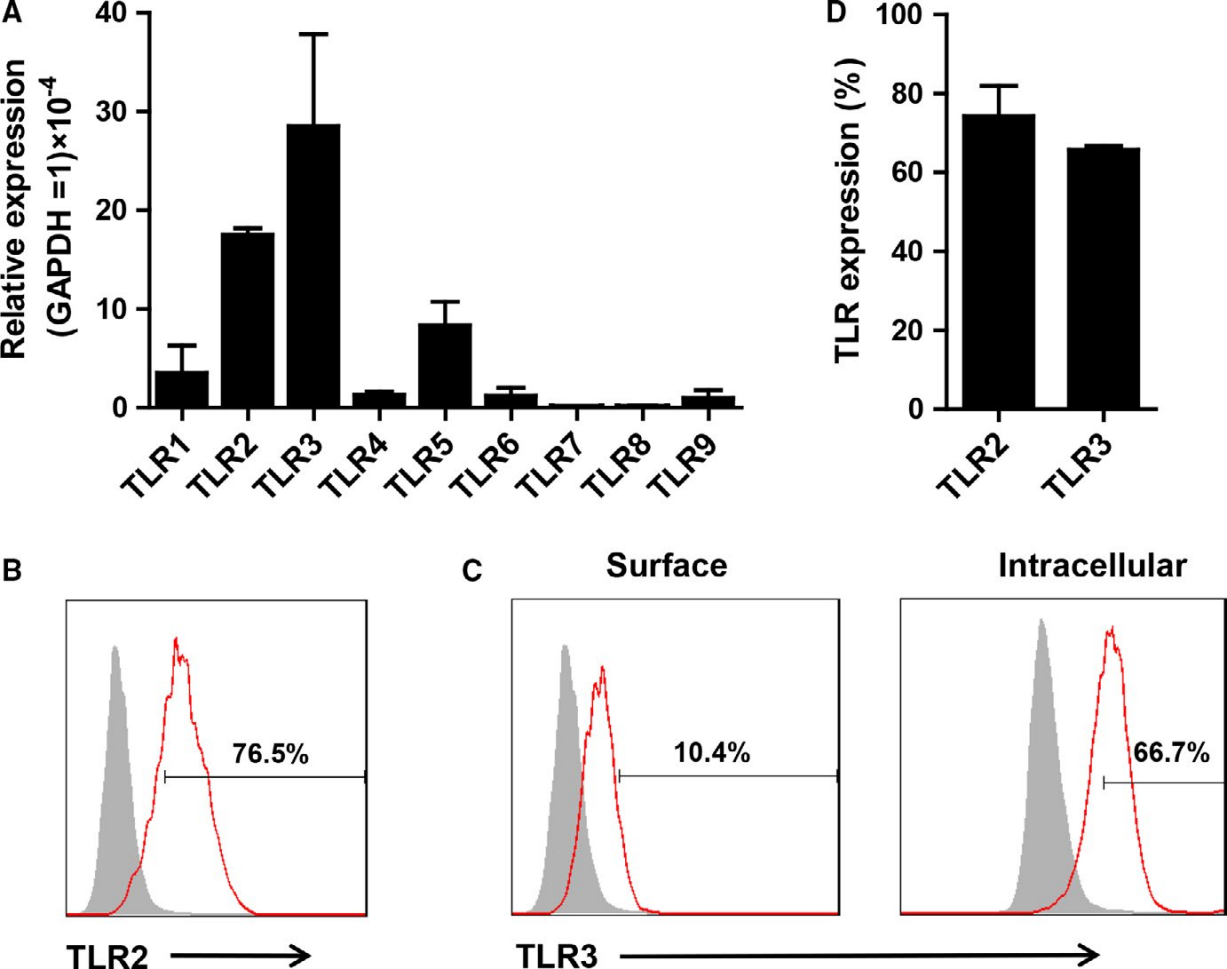
Expression of toll‐like receptors (TLRs) on telocytes. (A) The relative expression of TLRs was calculated against GAPDH. (B) The protein expression of TLR2 on the surface of telocytes was detected using flow cytometry. The filled lines indicated the isotype control. (C) The total protein expression of TLR3 on telocytes was detected using flow cytometry. The filled lines indicated the isotype control. (D) Statistical graph of TLR2 and TLR3 expression was shown

The TLR expression of telocytes was analysed using RT‐PCR and flow cytometry. Telocytes expressed TLR2, TLR3 and TLR5 at relatively high mRNA levels and TLR1, TLR4, TLR6, TLR7, TLR8 and TLR9 at lower levels (Figure 2A). On the contrast, cardiac fibroblasts expressed high levels of TLR2, TLR3 and TLR4 and lower levels of TLR1, TLR5, TLR6, TLR7, TLR8 and TLR9 (Figure S1). These data indicated that cardiac telocytes and fibroblasts had different patterns of TLRs. Then TLR2 and TLR3 expression were confirmed using flow cytometry. As expected, telocytes expressed abundant superficial TLR2 (approximately 80%) and intracellular TLR3 (approximately 70%) (Figure 2B‐D) and approximately 10% of TLR3 expression was observed on the cell surface. These data suggested that TLR2 and TLR3 might play important roles in regulating telocytes. According to the mRNA and protein expression of TLRs by telocytes, Pam3CSK4 (TLR1&2 ligand) and Poly I:C (TLR3 ligand) were used to stimulate telocytes in the following experiments.

### 3.3 The effect of TLR activation on telocyte proliferation and apoptosis

**Figure 3:**
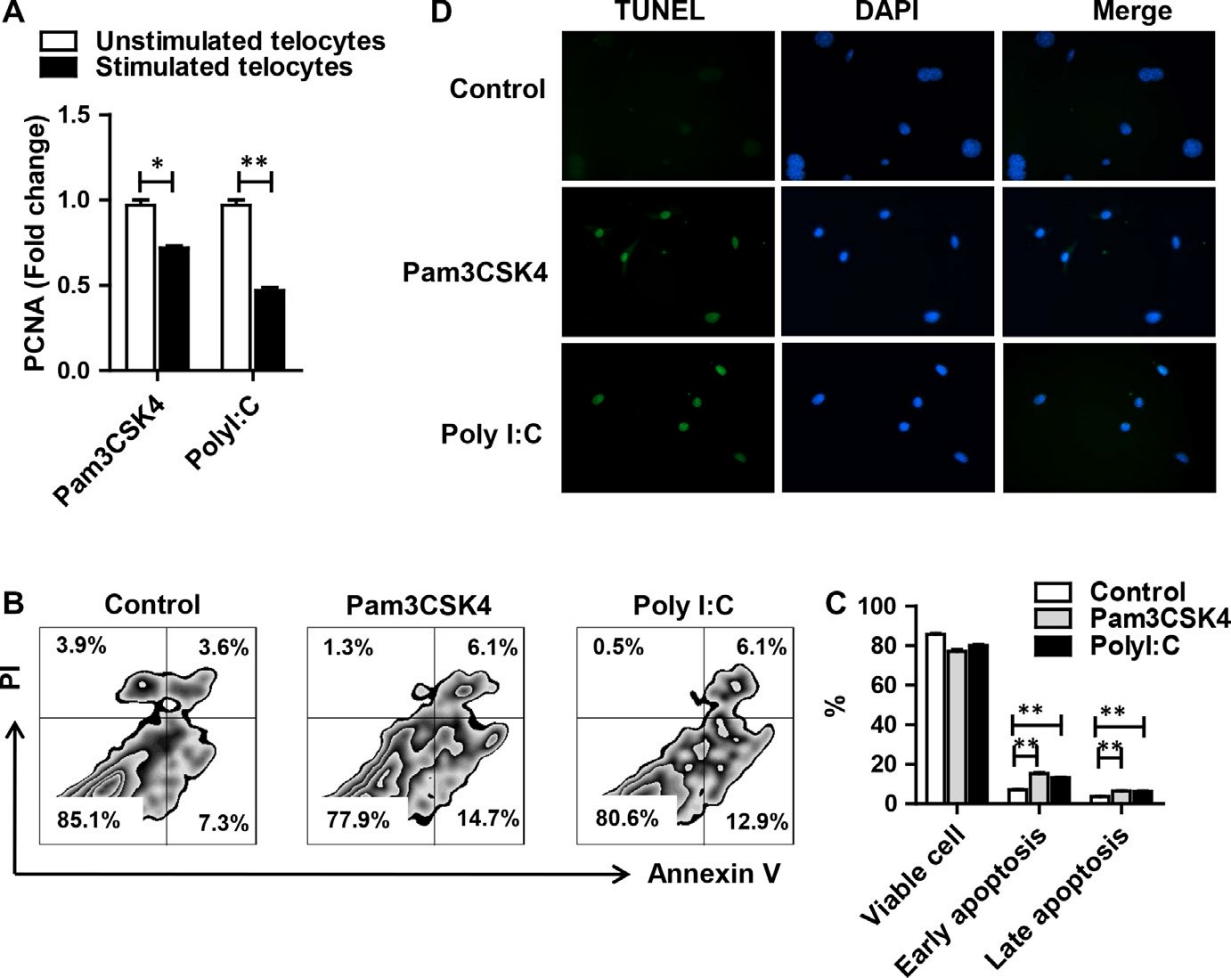
The effect of toll‐like receptor (TLR) agonists on proliferation and apoptosis of telocytes. (A) The PCNA expression of telocytes by RT‐PCR after TLR activation. (B) FACS analysis of Annexin V and PI in telocytes treated with Pam3CSK4 or PolyI:C for 24 hours, with medium alone as a control. Annexin V^+^PI^+^, Annexin V^+^PI^−^, Annexin V^−^PI^+^ and Annexin V^−^PI^−^ indicate late apoptotic, early apoptotic, necrotic and live cells respectively. (C) The percentage of cells labelled as Annexin V^+^PI^−^, Annexin V^+^PI^+^ and Annexin V^−^PI^+^ is shown in the histogram. (D) Images were shown for TUNEL staining after stimulation of TLR2 or TLR3 for 24 hours. Data are presented as mean ± SEM. *P < 0.05, **P < 0.01

Previous study showed that cardiac telocytes were reduced because of higher rates of cell apoptosis under heart failure.^30^ To investigate whether TLR had an effect on cell survival, telocytes were treated with TLR agonists for 24 hours. PCNA (proliferating cell nuclear antigen) was down‐regulated after TLR2 or TLR3 activation (Figure 3A). Apoptotic cells were positively stained with Annexin V or double positively stained with Annexin V and PI, whereas dead cells were only PI positive. Pam3CSK4 induced significantly higher apoptosis by Annexin V^+^PI^−^ (15.2%, early apoptosis) and Annexin V^+^PI^+^ (6.36%, late apoptosis) than that of the medium control group. PolyI:C treatment exhibited a comparable change (early apoptosis 13.15%, late apoptosis 6.19%) (Figure 3B,3). Moreover, we performed TUNEL staining to confirm our finding. As expected, stimulation of TLR2 or TLR3 could induce obvious apoptosis of telocytes (Figure 3D). These data indicated that TLR2 and TLR3 agonists could inhibit telocyte proliferation and promote apoptosis.

### 3.4 Telocyte secretory factors after TLR ligand stimulation

**Figure 4:**
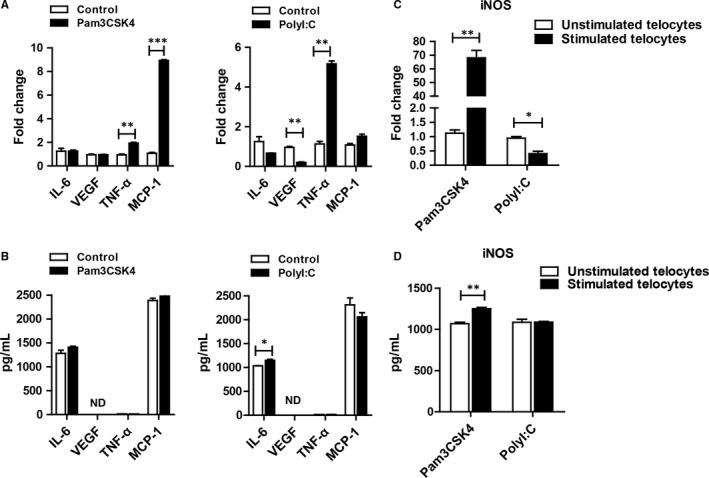
The cytokine production by telocytes could be regulated by Pam3CSK4 and PolyI:C. (A) The mRNA expression of IL‐6, VEGF, TNF‐α and MCP‐1 after stimulation for 24 hours with Pam3CSK4 or PolyI:C. (B) The expression of IL‐6, VEGF, TNF‐α and MCP‐1 was examined using ELISA after stimulation for 48 hours with Pam3CSK4 or PolyI:C. (C) iNOS mRNA expression after stimulation for 24 hours with Pam3CSK4 or PolyI:C. (D) The expression of iNOS was examined using ELISA after stimulation for 48 hours with Pam3CSK4 or PolyI:C. Data are presented as mean ± SEM. *P < 0.05, **P < 0.01, ***P < 0.001

As some groups described, telocytes could secret many factors, including cytokines, chemokines and extracellular vesicles.^11^ We wondered how the secretome changed after TLR ligand stimulation. IL‐6, VEGF, TNF‐α and MCP‐1 were selected as representative factors. For this purpose, telocytes were incubated with Pam3CSK4 or Poly I:C for 24 hours or 48 hours. Then, RT‐PCR and ELISA analyses were used to detect the expression of factors. TNF‐α and MCP‐1 were increased after TLR ligand stimulation. VEGF was down‐regulated after TLR3 activation, whereas it was not changed in the Pam3CSK4‐treated group when compared with the control group (Figure 4A). However, there were some discrepancies at the protein level (Figure 4B). These data suggested that TLR2 and TLR3 activation might induce different signalling pathways, resulting in diverse cytokine production. Unexpectedly, we found that telocytes could produce more abundant iNOS via TLR2 activation at the mRNA and protein levels compared with that via TLR3 activation (Figure 4C,4), which attracted our attention for further investigation.

### 3.5 PAm3CSK4‐pretreated telocytes suppress T cell activation through iNOS

**Figure 5:**
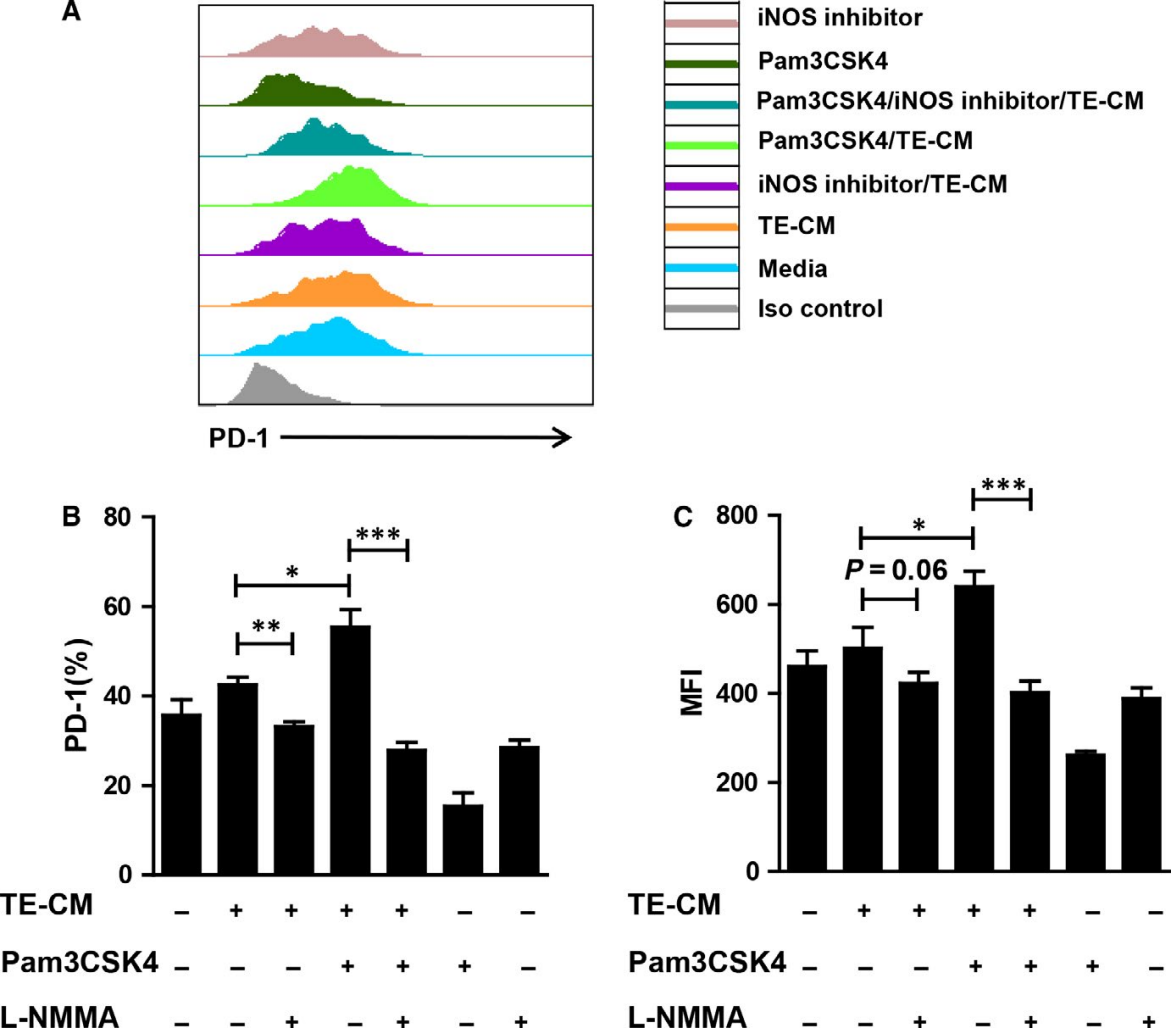
TLR2 enhanced the immunosuppression ability of telocytes by up‐regulating iNOS. (A) A representative histogram of PD‐1 expression on CD8^+^ T cells was shown. Splenocytes were co‐cultured with the supernatant of telocytes pre‐treated with or without Pam3CSK4 (3 μg/mL) for 4 days. An iNOS inhibitor (1.5 mmol/L) was added into some groups as indicated. (B, C) The PD‐1 percentage and MFI on CD8^+^ T cells were calculated as the treatment mentioned above. TE‐CM represents telocyte‐conditioned medium. Data are presented as mean ± SEM. *P < 0.05, **P < 0.01, ***P < 0.001

As we found above, Pam3CSK4 treatment could induce telocytes to produce large amounts of iNOS, which was reported to have immunosuppression ability. Therefore, we posited that telocytes could inhibit T cell activation. We performed co‐culture experiments to verify our hypothesis. Telocyte‐conditioned medium (TE‐CM) from telocytes pretreated with or without Pam3CSK4 and/or L‐NMMA for 24 hours was co‐cultured with activated spleen lymphocytes. After 4 days, PD‐1 expression was examined to evaluate the T cell state. Expectedly, TE‐CM slightly induced PD‐1 up‐regulation on CD8^+^ T cells. After TLR2 activation, telocytes acquired enhanced ability of immunosuppression (Figure 5A). In contrast, the addition of an iNOS inhibitor reversed the expression of PD‐1 on CD8^+^ T cells (Figure 5B,5). These data indicated that TLR2 activation could promote the production of telocyte iNOS, which was inhibitory to T cells.

## 4 DISCUSSION

Telocytes, a unique cell type, have gradually attracted our attention in the past 10 years. Previous studies focused on the morphology of telocytes^31^, ^32^ and their role in disease.^18^, ^33^, ^34^ However, the receptors on telocytes and their functions are still unknown. To our knowledge, this is the first study to demonstrate the expression of Toll‐like receptors and their immunoregulatory effects on telocytes. The results reported herein showed that TLR2 was dominantly expressed on telocytes among the TLR family and that its activation could enhance the immunosuppressive ability of telocytes.

The immunophenotype and receptors of telocytes were still not clear because of their short history. CD34 and PDGFR‐α were the most common markers for identifying telocytes in many organs.^29^, ^35^, ^36^ These markers indicated that telocytes might possess some features similar to stem cells and fibroblasts, although several groups had demonstrated differences.^37^, ^38^, ^39^ Vimentin and c‐kit were used to identify telocytes in the bovine teat sphincter and human lung.^40^, ^41^ Fei wang and colleagues compared various combinations of CD34, Vimentin, PDGFR‐α and PDGFR‐β.^42^ Double immunofluorescence by CD34/Vimentin could indicate more telocytes than CD34/PDGFR‐α and CD34/PDGFR‐β in a murine model of partial hepatectomy. However, not all the markers mentioned above must be used together to define telocytes. For example, telocytes isolated from the spleen were positive for Vimentin, CD34, Nanog and Sca‐1 but negative for c‐kit.^43^ These data suggested that there might be several subtypes of telocytes in different organs and their immunophenotype might be different depending on organs and animal species. Thus, morphological observation might be the best method to identify telocytes. Although some markers were explored to authenticate telocytes, there were few studies on receptors hinting at the function of the new cell type. Cretoiu CM^44^ reported that interstitial Cajal‐like cells expressed oestrogen and progesterone receptors, indicating their possible role in the female reproductive tract. Moreover, CD29, a marker of mesenchymal cells, was positive on telocytes, indicating the potential of telocytes to give rise to MSCs in culture.^37^ Telocytes were distributed in the intestine, liver, lung and heart, which were persistently exposed to microbiota, indicating their role in immune recognition. However, whether telocytes express pattern recognition receptors, such as TLRs, has not been clarified thus far. We demonstrated here initially that telocytes expressed high levels of TLR2 and TLR3 compared with other TLRs (Figure 2A2), indicating their possible roles in recognizing microorganisms. TLR2 and TLR3 are the first innate immune receptors found in the telocytes. These findings characterize the immunophenotype of telocytes, indicating that they will be regulated by the immune system. We observed increased cell apoptosis after TLRs activation (Figure 3). iNOS was reported as a driver of apoptosis.^45^ Indeed, our result showed that telocytes could produce a large amount of iNOS. These data suggested that TLRs induced apoptosis of telocytes via the iNOS pathway.

Telocytes were reported to be close to other cells in space, which led to the postulation that they might communicate with other cells. Recent studies have shown that telocytes participate in cell communication in two ways: (a) over long distances via telopodes and (b) over short distances via extracellular vesicles (EVs).^46^, ^47^ Telopodes and EVs might contain mitochondria,^48^ microRNA and many cytokines.^11^, ^49^ Wang's group performed proteomic analysis of lung telocytes and distinguished them from fibroblasts and endothelial cells by up‐regulated proteins, representing cellular functions such as intercellular communication and structure morphogenesis.^50^, ^51^ Furthermore, Laurentiu M. Popescu et al identified several proteins released from EVs.^11^ IL‐6, VEGF, MCP‐1 and CXCL1 were significant in mouse and rat telocyte supernatants and their concentrations were increased by passaging. We activated telocytes with TLR2 and TLR3 agonists in our research; surprisingly, there was a disparity in cytokine production because of the diverse pathways of activated telocytes, which needed to be further investigated (Figure 4A,4). In addition, telocytes contained measurable quantities of microRNAs (eg, let‐7e, miR‐27b, 126‐3p, 130a, 21, 22, etc).^16^, ^52^ These microRNAs played an important role in inflammation,^53^, ^54^ which suggested that telocytes might also participate in immunology. However, we did not focus on microRNAs in our study and how the microRNAs changed after TLR activation is still unknown.

Accumulating evidence has indicated that telocytes might develop cellular contacts with various cells, including dendritic cells, macrophages, mast cells, red blood cells, etc. For example, transmission electron micrographs showed that telocytes could connect with mast cells in the stromal synapse by its small cell body.^55^ In greater detail, Olga simionescu and colleagues demonstrated that telocytes maintained in close contact with mast cells, macrophages and mononuclear cells by their long telopode and an apoptotic telocyte with condensed chromatin has been found in the vicinity of a dendritic cell in psoriasis.^56^ Although a few studies have reported the function of telocytes,^57^, ^58^ little is known about the relationship between telocytes and other cells. We found that the level of iNOS was significantly increased after TLR2 activation (Figure 4C,4), suggesting that activated telocytes had enhanced the ability of immunosuppression. In vitro, we harvested supernatants of telocytes with different treatments as indicated to co‐culture with activated T cells. Telocytes stimulated by Pam3CSK4 could inhibit T cell activation. When we added L‐NMMA into the culture system, the immunosuppression state of T cells was reversed (Figure 5). These data suggested that activated telocytes could inhibit T cells via the iNOS pathway. Although our data revealed the possible role of telocytes in adjacent cells under stress conditions (TLR2 activation) for the first time, the downstream signalling pathway of telocytes regulated by TLR2 should be further explored.

The imbalance of apoptosis‐regeneration circle in cardiomyocyte induced by viral or bacterial infections contribute to heart injury. Telocytes were reported to involve in the regeneration of cardiomyocyte. We used the synthetic mimics of viral or bacterial to stimulate cardiac telocytes and found that activated telocytes were more likely to apoptosis and produce more cytokines. These data suggested that the apoptosis of telocytes might lead to the imbalance of cardiac cell composition, which could be one of the causes of heart disease. On the other hand, activated telocytes could secrete iNOS to avoid T cells over‐activation, indicating its role in maintaining cardiac homeostasis.

In conclusion, our present study demonstrated that telocytes expressed high levels of TLR2 and TLR3, the activation of which through ligands could modulate the proliferation, secretome and immunosuppression of telocytes. The findings indicated the potential application of telocytes in clinics to prevent T cell over‐activation. However, further studies should focus on the mechanisms that induce telocyte activation and mediate crosstalk between telocytes and other cells. Overall, our data filled in the gap by demonstrating the immunophenotype of telocytes and their function after TLR activation.

## STATEMENTS

All experiments were approved by the Jinzhou Medical University Animal Investigational Committee and were performed in accordance with the Guide for the Care and Use of Laboratory Animals published by the Ministry of Health of China.

## CONFLICT OF INTEREST

The authors declare that they have no conflict of interest.

## AUTHOR CONTRIBUTIONS

Conception and design: S. Li, X.S, S. He; Development of methodology: S. Li, S. He; Acquisition of data and revision of the manuscript: S. Li, XS; Analysis and interpretation of data: S. Li, XS; Writing the manuscript: S. Li, XS; Study supervision: S. He.

## DATA AVAILABILITY STATEMENT

The data that support the findings of this study are available from the corresponding author upon reasonable request
