# Supplementary figures and images for: Expression of toll‐like receptors and their regulatory roles in murine cardiac telocytes
Source: J Cell Mol Med. 2019 Jun 24;23(8):5360–8. doi: 10.1111/jcmm.14416 (PMC6653320; doi:10.1111/jcmm.14416)

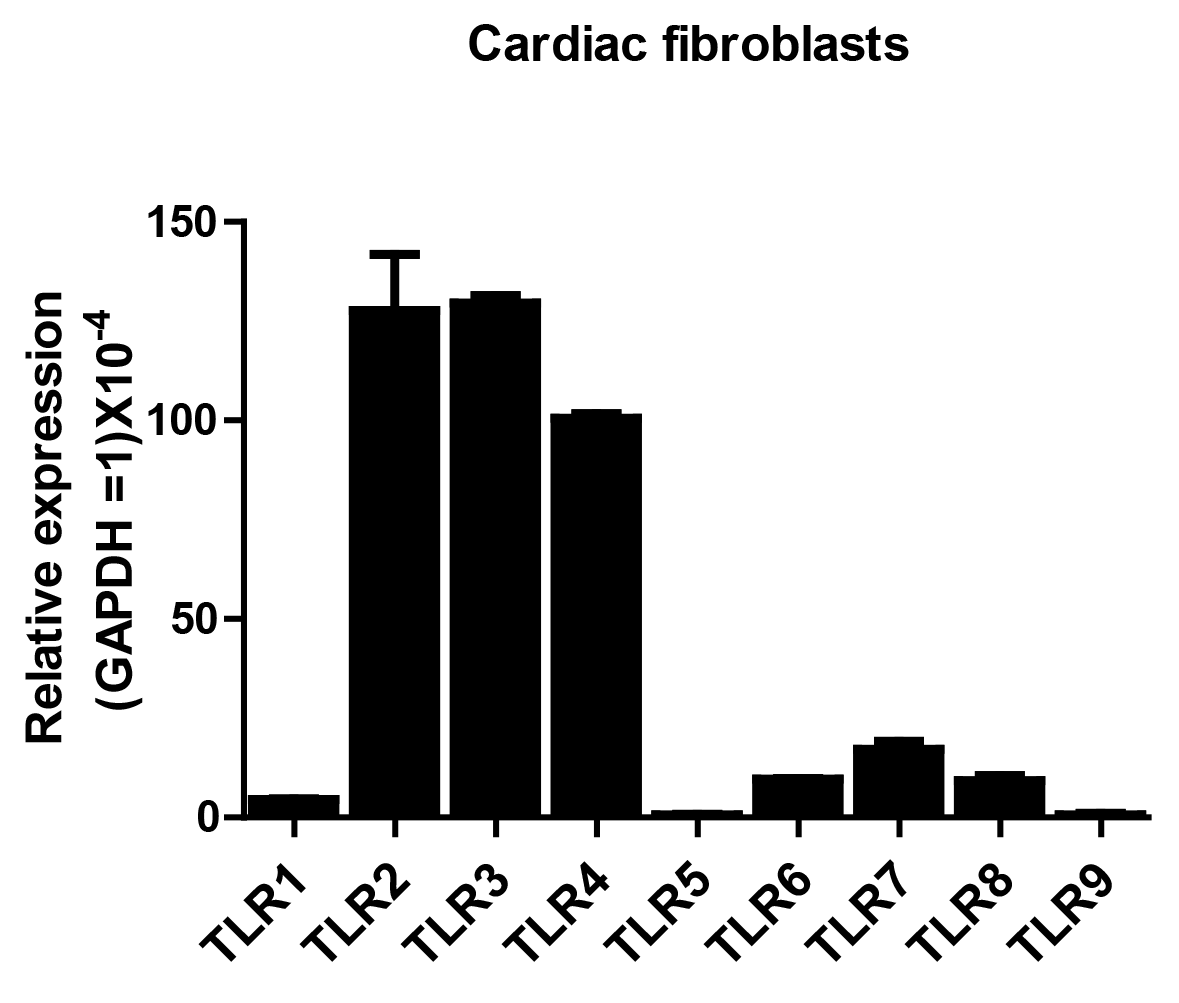

Supplement: Supplementary file 1 [file JCMM-23-5360-s001.tif]
